# SEA-NET: medical image segmentation network based on spiral squeeze-and-excitation and attention modules

**DOI:** 10.1186/s12880-024-01194-8

**Published:** 2024-01-11

**Authors:** Liangli Xiong, Chen Yi, Qiliang Xiong, Shaofeng Jiang

**Affiliations:** Department of BMC Medical Imaging, Nanchang Hangkong University, 330063 Nanchang, China

**Keywords:** Sample, Article, author

## Abstract

**Background:**

Medical image segmentation is an important processing step in most of medical image analysis. Thus, high accuracy and robustness are required for them. The current deep neural network based medical segmentation methods have good effect on image with balanced foreground and background, but it will loss the characteristics of small targets on image with imbalanced foreground and background after multiple convolutions.

**Methods:**

In order to retain the features of small targets in the deep network, we proposed a new medical image segmentation model based on the U-Net with squeeze-and-excitation and attention modules which form a spiral closed path,callled as Spiral Squeeze-and-Excitation and Attention NET (SEA-NET) in this paper. The segmentation model used squeeze-and-extraction modules to adjust the channel information to enhance the useful information and used attention modules to adjust the spatial information of the feature map to highlight the target area for small target segmentation when up-sampling. The deep semantic information is integrated into the shallow feature map by the attention model. Therefore, the deep semantic information cannot be scattered by continuous up-sampling. We used cross entropy loss + Tversky loss function for fast convergence and well processing the imbalanced data sets. Our proposed SEA-NET was tested on the brain MRI dataset LPBA40 and peripheral blood smear images.

**Conclusions:**

On brain MRI data, the average value of the Dice coefficient we obtained reached 98.1$\%$. On the peripheral blood smear dataset, our proposed model has a good segmentation effect on adhesion cells.

**Results:**

The experimental results proved that the proposed SEA-Net performed better than U-Net, U-Net++, etc. in medical image segmentation.

## Introduction

As medical image segmentation is the basic processing step in most of medical image analysis, medical image segmentation is required to have high segmentation accuracy and good stability. Therefore, it is necessary to develop automatic segmentation methods for medical images. However, traditional medical image segmentation methods cannot meet these requirements. Traditional automatic extraction methods need to adjust different parameters for different types of images, and can not be segmtioned across different types of images. Moreover, it is difficult to meet the needs of clinical and brain research in terms of extraction precision, extraction speed and extraction stability. The method proposed in this paper can solve these problems well. It can achieve high precision, fast and stable medical image segmentation without adjusting parameters. In recent years, the deep learning techniques have achieved rapid progress. Among them, the convolutional neural network (cnn) has great potential in the field of image processing due to automatic feature extraction, strong nonlinear expression ability, and no manual intervention. In the field of image segmentation, the common CNN models include FCN [1], PSP-Net [2] , mask-RCNN [3], U-Net [4] and so on. Among them, the extremely lightweight model U-Net is probably the most widely used model in medical image segmentation. U-Net has a simple structure, but its segmentation result is good. Many researchers chose it as the baseline to design model for various medical image segmentation. Cicek [5] extended the U-Net model to 3D image segmentation, and then applied it to brain tumor segmentation. Zhang [6] replaced each sub-block of U-Net with a staggered block, and then applied it to retinal segmentation. Zahangir [7] proposed R2U-Net that combined residual connections and cyclic connections to replace sub-blocks in U-Net. The improved model was verified on skin disease images and lung images. Oktay [8] proposed Attention U-Net that set attention gating between skip and connections to highlight more useful deep semantic features to compensate for low-level semantic features. However, If the feature of the small target is eliminated from the deep semantic features through multiple convolutions, attention to gating will not work on the low-level features of the small target. The output of the attention gated is still directly combined with the deep feature map, without considering the semantic gap between the shallow feature map and the deep feature map. Zhou [9] improved the skip-connection part and introduced deep supervision ideas based on U-Net. Huang [10] changed the skip connections into a full-scale skip connections. Jha [11] proposed Double U-Net structure. The first U-Net uses the pre-trained VGG-19 as the encoder, and the second U-Net uses ASPP to capture more information. It obtained good results on four different medical images. C. Guo [12] proposed spatial attention u-net and applied it to blood vessel extraction. M. Z. [13] applied U-net++ to the segmentation of brain tumors. Jieneng Chen [14] proposed TransUNet, which adopts hybrid CNN-Transformer and combines skip-connection to achieve better performance in medical image segmentation. A. Lin [15] proposes DS-TransUNet by using doubleSwin Transformer combined with U-shaped structure.

Unlike natural pictures, medical images usually have only one or two segmentation targets, and the proportion of target images is small. This kind of data is called category imbalance. If most of the training images are imbalanced images, the model may be able to learn the characteristics of small targets slowly. If there are both category imbalance and category balance images in training data, the model will tend to learn the characteristics of category balance. To obtain the global information of the big target, the network will be deepened. Multiple convolution operations will cause small target features to be lost. Since the shallow feature map contains the boundary information of the target and the global information of the small target, U-Net proposed a jump connection to combine the shallow feature maps from the encoding path and the deep feature maps of the same scale from the decoding path. However, simple jump connections cannot make full use of shallow feature maps. The deep semantic information is scattered after multiple upsampling operations. To solve these problems, we applied the Resnet based Squeeze-and-Excitation module ( SE-Res module) and attention module (A module ) into the U-Net, and proposed a new network model called SEA-Net. In SEA-Net, our main contributions are as follows:

- We replaced the copy-skip path with the attention path in U-Net model. Unlike Attention U-Net [8], this path is the only path that provides deep semantic information for the decoding. It combined deep semantics and shallow semantics to adjust the spatial information of the shallow feature map to highlight the target area, and provides more useful spatial semantic information for the decoding process.
- We added a SE-Res path parallel to the above attention path in the U-Net. The SE-Res path adjusted the channel information weight of the shallow feature map to remove redundant channel information, and to provide more channel semantic information for the decoding process.
- We proposed a hybrid function (cross entropy loss + Tversky loss) to handle the imbalance of data categories while ensuring that the model can still converge quickly.

## Methods

### Related work

*Attention mechanism* The attention mechanism in deep learning draws on the idea of human visual attention mechanism. It focuses on important points in a large amount of information, highlights key information, and ignores other unimportant information. The calculation of the attention mechanism can be divided into two steps. The first step is calculating the attention distribution on all information. The second step is calculating the weighted average of the input information according to the attention distribution. Various attention mechanism models have been proposed to tasks such as natural language processing [16], image classification [17], and machine translation [18]. Hu [19] proposed the Squeeze and Excitation module (SE), which performs attention operations in the channel dimension to allow the model to focus on the useful channel features for the task. Woo [20] proposed the CBAM module to capture global feature dependence based on spatial attention and channel attention. Wu [21] proposed a self-attention mechanism to capture the long and short distance relationship of pixels. Gao [22] proposed GCNet that combined the advantages of Non-local [23] and SENet [24]. GCNet [22] can effectively model the global context and is as lightweight as SENet. By calculating the relationship between two positions, the attention mechanism obtains long-range dependence and a wider receptive field, highlights the target area, and improves local constraints in the convolution process. While improving the performance of the model, the attention mechanism increases the interpretability of the neural network structure.

*Resnet* The deeper the network is, the richer the feature information we can obtain. However, simply deepening the network will cause problems such as network degradation and gradient disappearance. The residual network (Resnet) [24] was proposed to make the deep network can be well trained. It can increase the accuracy of the model by adding considerable depth. The residual block inside the residual network uses skip connections, which alleviates the problems of network degradation and gradient disappearance caused by the deepening of the deep neural network. The idea of skip connect is to express the output as a linear superposition of a nonlinear transformation of the input and the output. Specifically, skip connection protects the integrity of the information by directly detouring the input information to the output. The entire network only needs to learn the difference between input and output. It simplifies learning objectives and difficulty. The residual block has a simple structure and is optimized easily, so it is often embedded in various deep convolutional networks. Diakogiannis [25] proposed ResUNet-a for remotely sensed data; Ibtehaz [26] replaced the simple skip connection in traditional U-Net with residual path. Resnet has two structures, one is concatenated by two 3$\times$3 convolutional layers; the other is concatenated by three convolutions of kernel size 1$\times$1, 3$\times$3, and 1$\times$1. Researchers will choose the appropriate resdual block structure according to the requirements in the task.

*Category imbalance* In deep learning, most data sets have the problem of category imbalance. In medical image segmentation, the imbalance between foreground and background is particularly serious. There are only one or two targets in a image, and the pixel ratio of the targets is relatively small, which will make it difficult to train the network. Some researchers tried to use a reasonably designed loss function to achieve accurate segmentation of small targets. Field [27] proposed Dice loss to increase the weight of the foreground area. Seyed Sadegh [28] proposed a generalized loss function of Tversky exponent to find a better balance between accuracy and recall. The improved loss function can solve the problem of data imbalance while retaining all the information of the image.

### Methodology

**Fig. 1:**
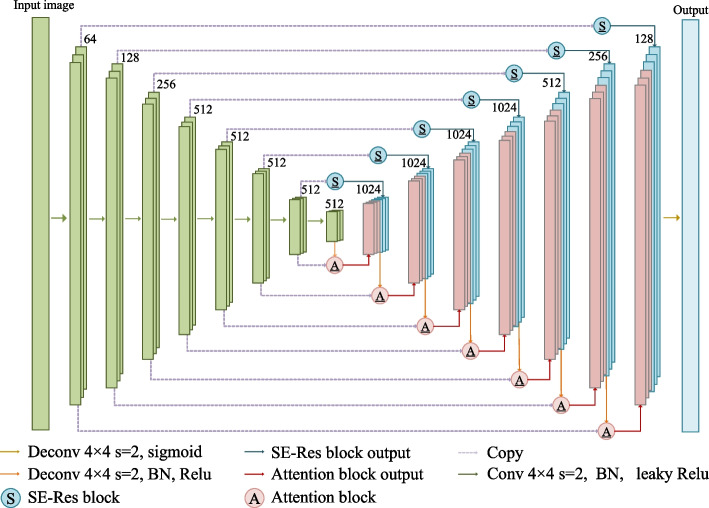
The overall architecture of the SEA-Net model. In the figure, A represents the attention module, and S represents the compression excitation module. They form a closed spiral path between the encoder and decoder in U-Net

The structure of the proposed segmentation model is an encoding- decoding architecture. The overall network architecture is shown in Fig. 1. We will introduce the proposed SEA-Net model from encoder, decoder, and two skip paths.

*Encoder* The left side in Fig. 4 is the encoder. The encoder has 8 convolutional components including 4$\times$4 convolutional layer, BN and leaky Relu activation layer to provide wider receptive field. Since the pooling operation will cause loss of feature information, we use a convolution operation with stride of 2 to achieve downsampling. Both shallow information and deep information are important for medical image segmentation. We hope to obtain more feature maps of different scales to improve the segmentation performance of the model. Therefore, the number of convoluation layer of our encoder is twice that of the original U-Net network. The encoder obtains multi-scale feature information through layer-by-layer convolution. The shallow feature map has insufficient receptive fields, but has more detailed information of image such as the characteristics of small target and the boundary of the target. The deep feature map has less detailed information of image but has a wide receptive field, so it is important for object positioning. Therefore, the decoder needs to better integrate multi-scale feature maps.

*Decoder* The right side in Fig. 1 is the decoder. The encoding-decoding architecture is a symmetrical structure. After the encoder is down-sampled 8 times, the decoder needs to go through 8 up-sampling to restore the image resolution. The up-sampling process of the original U-Net is that the deep feature map first achieves scale expansion through an up-sampling operation such as nearest field interpolation, and then combines the corresponding scale feature map from the encoding path as the input of the next up-sampling operation.

There are semantic dissimilarities between deep feature maps and shallow feature images, and the deepened network loses a lot of image details. Simple skip connection cannot solve these problems well. The proposed SEA-Net can solve the above problems by redesigning two parallel skip paths in the decoder.

**Fig. 2:**
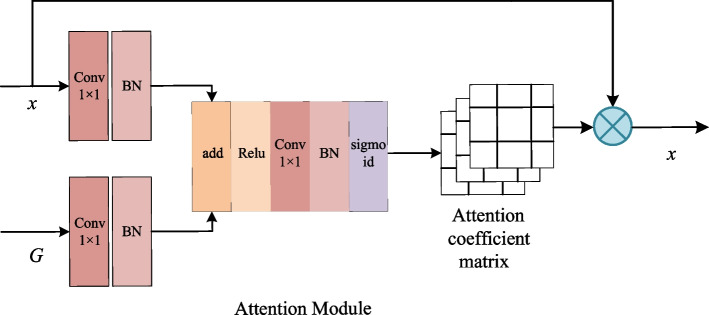
The internal structure diagram of the attention module

**Fig. 3:**
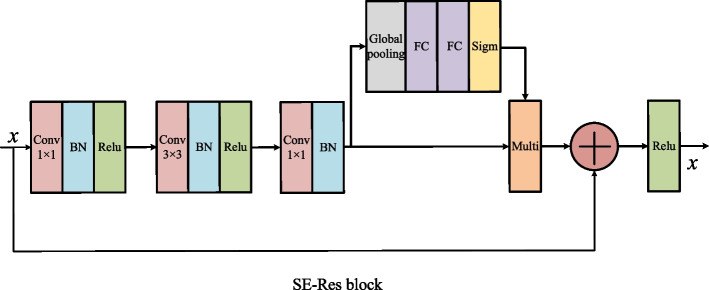
The internal structure diagram of the SE-Res path. The SE module is located between the last BN output and the shortcut connection

Figure 2 shows the detailed internal structure of the attention block in Fig. 2. Figure 3 shows the detailed internal structure of the SE-Res module in Fig. 1. The attention module and the SE-Res module of the same level has the same input. The G in Fig. 2 corresponds to the deep feature map, and x corresponds to the shallow feature map. Before going into the attention block, the deep feature map first is expanded through deconvolution with kernel size of 4$\times$4 and stride of 2 and becomes the input G of the attention path. Then G with deep semantic information guides the feature map x at the same level from the coding path to redistribute feature weights through the attention path. x is also used as the input of SE-Res path to adjust the channel information weight through the SE module. Finally, the output $\dot{X}$ of the attention path is concatenated to the output $\ddot{X}$ of the SE-Res path to generate the input G of the next attention path. This structure not only ensures that the shallow feature map and the deep feature map are not directly connected to avoid the feature fusion of semantic gap in the U-Net, but also ensures that features of the small targets lost by attention module can be recovered by adding the shallow feature map back. Thus, the semantic information of small targets is not diluted by accumulated convolutional neural operations, and at the same time it can make full use of the multi-scale feature information to retain more shallow semantic information.

*Attention path* The attention path we added is not only used for deep semantic integration into the decoding path, it can also suppress noises in medical images. It used deep semantic information to adjust the weight ratio of the target area and non-target area, highlights the target area, and provides more useful feature information to the model.

In the Fig. 2, x and G first go through a convolutional layer with a convolution kernel of 1$\times$1 followed by a BN layer for channel number adjustment and cross-channel feature integration. Next, the two feature maps are superimposed to highlight the objects of model learning followed by a Relu activation function to suppress the non-target region. Then the value of the attention coefficient is concentrated between 0 and 1 through the convolution layer, BN layer and the sigmoid activation function layer. Finally, the obtained attention coefficient matrix is multiplied with the original x to output a new feature map with adjusted weights.

*SE-Res path* The internal structure of SE-Res path is shown in Fig. 3. The SE module is embedded in residual block. The residual block is divided into convolution paths and shortcuts connection. The convolution path consists of 3 convolution blocks with convolution layer, batch normalization layer, and activation function Relu layer. The SE module is added between the BN layer of the third convolution block and the shortcut connection.

The SE module is proposed to focus on the relationship between feature channels. The model automatically learns the importance of different channel features through the SE block. The squeeze operation is to calculate the average of all information on the two-dimensional feature map of each channel through the global average pooling layer. Squeeze operation can eliminate the interference of spatial information. The extraction operation is implemented by two full connections (FC) and one Sigmoid activation layer. The first FC layer compresses n channels into n/8 channels to reduce the calculation. The second FC layer restores n channels. The weight coefficient of each channel is trained by FC using channel correlation. The output of the sigmoid layer is multiplied to the output of last BN in Resblock.

Since there is a semantic gap between the shallow feature map of the encoder and the corresponding deep feature map of the decoder, we did not set the SE block separately on the skip connection. The shortcut connection adds the input feature map x to the output of SE, and finally activates it through the Relu layer.

### Training

*Loss function* The most commonly used loss function in medical image segmentation is the pixel-by-pixel cross-entropy loss. Researchers use a cross-entropy loss function to measure the gap between the predicted mask output by the model and the true label. The expression is as follows:

1 $$\begin{aligned} L_{C E}=-\frac{1}{\mathrm {~m}} \sum \limits _{i=1}^m \sum \limits _{j=1}^n p\left( x_{i j}\right) \log \left( q\left( x_{i j}\right) \right) \end{aligned}$$

Where m represents the number of samples, $p\left( x_{i j}\right)$ represents the category of the pixel, and $q\left( x_{i j}\right)$ represents the probability of the corresponding category of the pixel. The cross-entropy loss function evaluates the class prediction of each pixel individually, and then averages all pixels. All pixels in the image are learned indiscriminately. However, the problem of imbalanced categories often occurs in medical images, that is, the proportion of target pixels is much smaller than that of non-target areas. Only using the cross-entropy function as the loss function will result in biased training towards the category with more pixels. For small objects, it is difficult for the model to learn its characteristics, which reduces the effectiveness of the network. In order to solve the problem of data imbalance, we added Tversky loss on the basis of cross entropy loss. Tversky coefficient is a generalized function of Dice coefficient and Jaccard coefficient. The formula is as follows:

2 $$\begin{aligned} L_{T(A, B)}=\frac{|A \cap B|}{|A \cap B|+\alpha |A-B|+\beta |B-A|} \end{aligned}$$

When $\alpha =\beta =0$, the Tversky coefficient is the Dice coefficient. When $\alpha =\beta =1$, the Tversky coefficient is the Jaccard coefficient. $|A \cap B|$ means false positive, $|B \cap A|$ means false negative. $\alpha$ and $\beta$ can control the trade-off between false positives and false negatives.

Since the cross entropy will be much smaller than Tversky loss after several rounds of training, the total loss function of this model is:

3 $$\begin{aligned} L=L_{C E}+\lambda \times L_T \end{aligned}$$

$\lambda$ can be adjusted as needed. We take 10 on the brain MRI dataset LPBA40.

*Implementation details* The optimizer we chose is adam, where beta1 is 0 and beta2 is 0.9. The learning rate parameter is set to 0.0001. The brain MRI dataset LPBA40 is trained for 10 epochs, and the blood smear data set is trained for 50 epochs. The training time of this model on an NVIDIA gtx 1080Ti GPU ranges from 1 hour to 3 hours.

### Experiments

*Datasets* Our model was validated on two datasets. One is the public T1-weighted brain MRI data set LPBA40 for brain tissue extraction. LPBA40 dataset was obtained on the LONI website of the University of Southern California Los Angeles, and its spatial resolution is 0.86$\times$1.5$\times$0.86. The dataset has 40 normal human brain scan data. The ratio of the number of training sets to the number of test sets in this data set is 3: 1. The other data set is a self-built blood smear image data set for white blood cell extraction. There are 316 blood smear images with spatial resolution of 640$\times$800. Each image contains 1-10 white blood cells. In the dataset, 19 blood smear images including adhesion cells are used as test set A. The remaining images are divided into training set and test set B, the ratio of which is 3: 1.

*Evaluation metrics* In order to evaluate the performance of the model, three evaluation indicators need to be calculated in the image segmentation task. They are Dice coefficient, sensitivity, and specificity. Dice coefficient was used to measure the similarity between prediction mask and label:

4 $$\begin{aligned} Dice =\frac{2 T P}{2 T P+F P+F N} \end{aligned}$$

Sensitivity represents the ability of segmentation methods to correctly recognize target area:

5 $$\begin{aligned} sensitivity =\frac{T P}{T P+F N} \end{aligned}$$

Specificity represents the ability of the methods to correctly recognize non-target area.

6 $$\begin{aligned} specificity =\frac{T N}{T N+F P} \end{aligned}$$

where TP is true positive, FP is false positive, and FN is false negative. Values of Dice coefficient, sensitivity, and specificity range from 0 to 1. The larger the values of these three evaluation coefficients are, the more accurate the segmentation results are.

Accuracy is the proportion of correct predictions in all predictions.

7 $$\begin{aligned} Accuracy =\frac{T P+T N}{T P+F N+F P+F N} \end{aligned}$$

## Results

Table 1 lists the experimental evaluation results of various brain segmentation methods on LPBA40. Besides the methods we proposed, the methods to participate in the comparison experiment include the baseline method (U-Net [4]), the state-of-the-art brain segmentation method (Attention U-Net [8], U-Net++ [13], and 3D U-Net [5]) and the classic brain segmentation method (BET [29], ROBEX [30]). On LPBA40, the Dice coefficient value of the classic brain segmentation method BET is the lowest among the methods participating in the comparison. Although the Dice coefficient value of another classic segmentation method ROBEX ranks fourth among these methods, its sensitivity-specificity is not balanced. The training time of U-Net++ and 3D U-Net is 5 times that of U-Net, but their performance is lower than its baseline model U-Net on LPBA40. The Dice coefficient value of Attention U-Net is lower than baseline U-Net. Therefore, simply adjusting the spatial of the coding matrix will not effectively improve the segmentation task. Attention U-Net does not solve the semantic gap generated by the direct combination of deep feature maps and shallow feature images. By contrast, the attention path added in SEA-Net retained both deep and shallow features in the image, thus SEA-Net obtained the highest Dice coefficient value and specificity value in the data set LPBA40, and the sensitivity also ranked second in the automatic brain extraction lgorithm comparison experiment.

**Table 1:** The mean of the three evaluations of the public dataset of LPBA40 with different brain extraction methods

Methods	Dice($\%$)	Sensitivity($\%$)	Specificity($\%$)
SEA-Net	**98.15**	98.40	**99.63**
U-Net	97.51	99.43	99.17
Attention U-Net	97.47	98.24	99.38
U-Net++	96.55	**99.82**	98.73
3D U-Net	96.16	98.11	98.93
BET	92.01	98.97	96.82
ROBEX	96.74	96.22	99.49

**Fig. 4:**
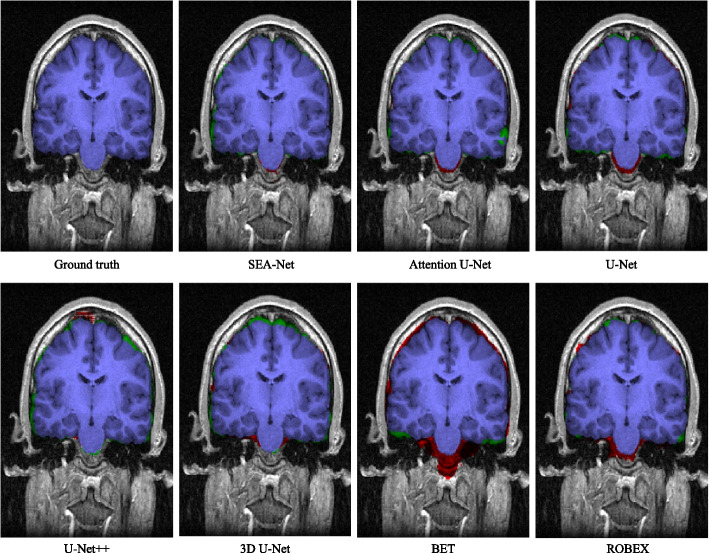
The overlay mask map of each segmentation algorithm participating in the comparison experiment. Red represents over-segmentation and green represents under-segmentation. The original image is from a slice of the coronal section of the 23rd case in the data set LPBA40

In Fig. 4, we can see that our segmentation results are very close to ground truth. SEA-Net also can accurately obtain the boundaries of brain tissue. Other brain segmentation methods have more serious over segmentation and under segmentation.

**Fig. 5:**
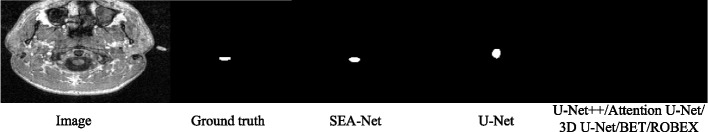
The output mask of one of the slices in the 23rd case of LPBA40 and their corresponding input images and labels for each method participating in the comparison experiment

**Fig. 6:**
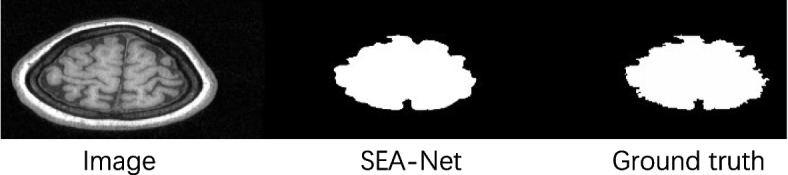
One of the poorly segmented images in the LPBA40 dataset. It can be seen that for images with jagged edges, SEA-NET segmentation is too smooth, and some tiny brain tissue models are not segmented

The dataset LPBA40 we used are 2-dimensional slices of 3-dimensional brain MRI images. Therefore, part of the brain MRI image needs to be segmented in a very small brain tissue area. This type of picture is shown in Fig. 5. The target area occupies a very small proportion in the entire image, so the target information is easily lost. In Fig. 5, only the SEA-Net model we proposed and U-Net model segment the brain tissue successfully, the other methods output a completely black image, and U-Net obviously led to over-segmentation. In Fig. 6, it can be seen that the SEA-NET segmentation is too smooth.

SEA-Net has a very good performance on the data set LPBA. The model is proved to be able to deal with the imbalanced images in LPBA40 well. Since the LPBA40 data set contains both category-balanced images and category-imbalanced images, in order to eliminate the interference of category-balanced images and determine its segmentation effect on small targets, we also chose a data set with imbalanced categories in medical images (the peripheral blood smear image). The peripheral blood smear image dataset has a high resolution, but the segmented target white blood cells are small in both size and number, and there are many adhesion cells.

**Table 2:** Comparative experiments on white blood cell extraction from the peripheral blood smear images

Methods	Dice($\%$)	Sensitivity($\%$)	Specificity($\%$)	Accuracy($\%$)
U-Net	80.14	79.33	**99.92**	99.83
Attention U-Net	80.04	79.80	99.90	98.82
U-Net++	80.69	81.53	99.53	**99.84**
SEA-Net	**82.35**	**90.74**	99.72	99.83

**Fig. 7:**
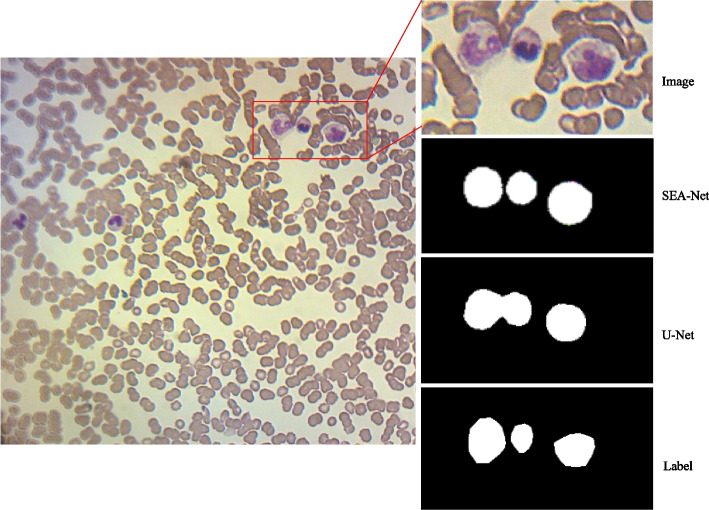
The blood smear image, the magnified adhesion cell area and the corresponding mask map obtained by various segmentation methods

Table 2 lists the experimental evaluation results of peripheral blood smear images by various methods. In addition to our proposed method, other methods involved in the comparison experiment include the baseline method (U-Net [4]), the most advanced cell segmentation method (Attention U-Net [8], U-Net ++ [10]). We also added an accuracy measure to show whether the number of white blood cells dividing is correct. SEA-Net’s Dice coefficient improved by more than 2$\%$ over the baseline model and the state-of-the-art U-Net++ and Attention U-NET models. We observed that in many blood smear images, there were adherent cells or cells clustered together, and both Attention U-Net and U-Net ++ + incorrectly identified the two units near each other as one unit, which should be the cause of the reduced accuracy of Attention U-Net and U-Net ++ models. The model we proposed can solve this problem well. In addition, compared with the most advanced UN ++ and U-Net, which is currently the most concerned, the specificity of SEA-Net is slightly reduced, but the sensitivity is increased by about 10$\%$, achieving a balance of specificity and sensitivity.

Figure 7 shows them in a large-resolution image, our proposed method can well complete the segmentation of adhesion cells due to the excellent ability to retain both the shallow image features (boundary of the blood cell) and the deep semantic features (target of the blood cell). By contrast, the U-Net focused on rather the deep semantic feature than the shallow image features, therefore, the boundary between the adhesion cells were blurred, leading to wrong detection.

**Table 3:** Results of ablation experiments with different methods on LPBA40 dataset

Methods	Dice($\%$)	Sensitivity($\%$)	Specificity($\%$)
Baseline	97.51	99.43	99.17
Baseline+SE-Res	97.67	98.60	99.40
Baseline+Attention	97.47	98.24	99.38
Baseline+Attention+hybrid function	97.69	97.97	99.51
Baseline+hybrid function	97.55	97.71	99.55
Baseline+SE-Res+hybrid function	97.82	98.49	99.46
Baseline+SE-Res+Attention	97.72	**98.67**	99.36
Full	**98.15**	98.40	**99.63**

**Table 4:** Experimental results of different ablation methods on peripheral blood smear image dataset

Methods	Dice($\%$)	Sensitivity($\%$)	Specificity($\%$)
Baseline	80.14	79.33	99.92
Baseline+SE-Res	81.61	82.90	99.91
Baseline+Attention	80.04	82.56	99.90
Baseline+Attention+hybrid function	80.13	79.08	**99.92**
Baseline+hybrid function	80.08	80.92	99.91
Baseline+SE-Res+hybrid function	82.28	84.41	99.84
Baseline+SE-Res+Attention	80.89	81.13	99.83
Full	**82.35**	**90.74**	99.83

In order to verify the effectiveness of attention path, SE-Res path and hybrid function, we made an ablation study. Table 3 liststhe evaluation indexes of ablation experimental mean values of different networks on LPBA40 data sets. The bold values in the table indicate the best results.

Attention path, SE-Res path, and hybrid function have all improved dice and specificity for the baseline specificity, of which the mixed loss function has the best effect. It was proved that the three modules could enhance dice and Specificity of the baseline model. All three modules have decreased Sensitivity to different degrees, and SEA-Net can alleviate the decrease of Sensitivity index.

Table 4 lists the evaluation indicators of the mean value of different network ablation experiments on the peripheral blood smear image dataset. Compared with the baseline model, each module has different degree of improvement on the dice and brings similar improvement effects. Both sensitivity and specificity were decreased to varying degrees. SEA-Net improves the end result, balancing specificity and sensitivity.

## Conclusion

In this paper, we proposed an extended U-Net model called SEA-Net, using squeeze and extended model and attention model. This model was applied to two different medical image data sets including the brain MRI data set LPAB40 and the peripheral blood smear data set. On the data set LPBA40, the experimental results proved that the proposed SEA-Net performed better than U-Net, U-Net++, etc. in medical image segmentation. On the peripheral blood smear data set, SEA-Net proved the applicability and accuracy of small target segmentation. In the future, we will explore 3D small target segmentation tasks.

## Data Availability

The datasets analyzed in this study are available from the corresponding author on reasonable request.
